# Optimizing fixed-time laparoscopic artificial insemination in the jaguar

**DOI:** 10.1530/RAF-25-0074

**Published:** 2026-01-29

**Authors:** Lindsey Marie Vansandt, Cristina Harumi Adania, Priscila Rocha Yanai, Jéssica da Silva Paulino, Regina Celia Rodrigues da Paz, Helen L Bateman, Elizabeth Marie Donelan, William F Swanson

**Affiliations:** ^1^ Center for Conservation and Research of Endangered Wildlife, Cincinnati Zoo and Botanical Garden, Cincinnati, Ohio, USA; ^2^ Centro Brasileiro Para Conservação dos Felinos Neotropicais, Associação Mata Ciliar, Jundiaí, SP, Brazil; ^3^ Laboratório de Pesquisa em Animais de Zoológico, Faculdade de Medicina Veterinária, Universidade Federal de Mato Grosso, Cuiabá, MT, Brazil

**Keywords:** altrenogest, artificial insemination, assisted reproductive technologies, equine chorionic gonadotropin, porcine luteinizing hormone

## Abstract

The jaguar faces significant threats to survival from habitat fragmentation/loss and poaching. Conservation efforts include maintaining *ex situ* populations in zoos, but breeding success remains limited, necessitating the development of assisted reproductive technologies. This study aimed to optimize ovarian synchronization and artificial insemination (AI) protocols for jaguars by evaluating ovarian follicular activity during oral altrenogest treatment and in response to equine chorionic gonadotropin (eCG)/porcine luteinizing hormone (pLH) regimens, and assessing fertility outcomes with laparoscopic AI. In experiment 1, females were fed altrenogest (0.044 or 0.088 mg/kg, *n* = 2 per dose) daily for 45 days, but neither dose suppressed ovarian activity. In experiment 2, females received 0.088 or 0.176 mg/kg altrenogest (*n* = 2 per dose) for 45–48 days and then the alternate dose six months later. After a 5-day withdrawal, 600 IU eCG and 5,000 IU pLH (82 h interval) were administered intramuscularly. All eight cycles produced multiple follicles, but ovulation rates were low and no pregnancies occurred. In experiment 3, five females received 0.176 mg/kg altrenogest, followed by a 7-day withdrawal, 600 IU eCG, and 10,000 IU pLH (90–92 h interval). All females achieved multiple ovulations, and one female conceived, delivering a live cub after 103 days – the first jaguar ever produced from AI. In summary, altrenogest and eCG/pLH treatment following species-specific adjustments was effective for ovarian synchronization in the jaguar. The birth of a live cub following AI validates the effectiveness of this hormone regimen and highlights the potential of AI for population management of this imperiled felid.

## Introduction

The jaguar (*Panthera onca*) is the largest wild cat native to the Americas and a focal species for conservation efforts. Historically distributed from the southwestern United States through southern Argentina, jaguars have experienced significant declines in both range and population size due to habitat loss/fragmentation and poaching (Quigley *et al.* 2017). As a result, jaguars are now classified as near-threatened on the International Union for Conservation of Nature’s Red List of Threatened Species (Quigley *et al.* 2017).

There is a large *ex situ* jaguar population (>500 individuals) housed under human care in Brazil and other Latin American countries, as well as in North American and European zoos (da Paz *et al.* 2023). These cats serve as an assurance population that can protect against species extinction and potentially allow reintroduction of zoo-born animals back into the wild. Although reintroduction programs are still limited, early efforts, such as those in the Iberá Wetlands of Argentina, have shown successful establishment and reproduction of released zoo-born jaguars (Donadio *et al.* 2022). Zoo-housed cats also act as ambassadors for their *in situ* counterparts, helping to educate guests and communicate the messages of conservation to broad audiences. Finally, cats in zoos also provide scientists with the unique opportunity to more readily study their behavior and physiology.

In North American zoos accredited by the Association of Zoos and Aquariums (AZA), the Felid Taxon Advisory Group (TAG) is responsible for the management of all non-domestic felids within formal breeding programs called Species Survival Plans® (SSPs). The current Jaguar SSP population consists of 77 individuals housed in 44 AZA institutions, with 57 (30 males and 27 females) designated as part of the pedigreed breeding population (Johnson *et al.* 2024). The goal of the Jaguar SSP is to establish a sustainable population of 90 individuals while maintaining at least 90% genetic diversity over the next 100 years. Although the SSP experienced periods of growth in the past due to managed breeding programs, recent years have seen a marked decline in reproductive success. This is compounded by the aging demographic structure, with many individuals reaching post-reproductive age, and limited success among younger breeding pairs. Despite annual recommendations for 10 to 13 breeding pairs, only a limited number successfully produce cubs, with the population averaging just one birth per year from 2018 to 2024 (Johnson *et al.* 2024).

Brazil’s *ex situ* jaguar population is managed through a collaborative effort between the Association of Zoos and Aquariums of Brazil (AZAB) and the Chico Mendes Institute for Biodiversity Conservation (ICMBio). This partnership focuses on maintaining genetic diversity and ensuring the health and well-being of jaguars housed under human care. As of 2021, Brazil’s *ex situ* jaguar population consists of 82 individuals (32 males and 50 females) across 34 institutions (da Paz *et al.* 2023).

Jaguars in Brazilian zoological institutions have adapted well to human care and often reach advanced ages. However, natural breeding efforts have had limited success (da Paz *et al.* 2023). Many institutions face challenges in housing more than one breeding pair, leading to situations where animals that have failed to reproduce remain until they die or are transferred. As a result, many institutions are left with aging animals that can no longer contribute to breeding programs. AZAB/ICMBio regularly provides recommendations for new pairings and management strategies. Unfortunately, the jaguar’s large size and aggressive nature, combined with financial, logistical, and safety challenges, often make animal transport difficult and hinder the implementation of these recommended pairings (da Paz *et al.* 2023).

Due to these challenges, natural breeding alone does not produce enough births in Brazilian zoos to maintain a stable population. Instead, the genetic and demographic health of the *ex situ* population has largely relied on the intake of wild-born individuals, especially orphaned cubs from the Amazon biome. Notably, 51 of the 82 jaguars in the current *ex situ* population originated from the Amazon (da Paz *et al.* 2023).

There is a critical need to improve reproductive success in jaguars to meet demographic goals and stabilize the *ex situ* populations. One means to address these challenges is through the development of assisted reproductive technologies (ARTs). Artificial insemination (AI) represents one of the most readily applicable ARTs in felids, as it can overcome behavioral incompatibilities in breeding pairs, eliminate the need for animal transport between zoos, and facilitate the introduction of new genetics from founders without removing animals from the wild (Pukazhenthi & Wildt 2004). While AI has been used to successfully propagate 14 wild felid species (reviewed by Swanson (2023)), no successful AI has been reported in the jaguar.

This study aimed to develop an optimized ovarian synchronization protocol for AI in jaguars, integrating treatment with oral progestin to suppress ovarian activity and exogenous gonadotropins (i.e., equine chorionic gonadotropin, eCG; porcine luteinizing hormone, pLH) to stimulate follicular development and ovulation, respectively. In particular, our objectives were to i) evaluate ovarian sensitivity to oral progestin, ii) examine ovarian responses to exogenous gonadotropin treatment, and iii) assess fertility outcomes using fixed-time laparoscopic oviductal and/or uterine AI. Progestin suppression and eCG/pLH stimulation regimens were selected based on their demonstrated efficacy in inducing timed ovulation in other felid species, with the expectation that species-specific modifications would be required. Laparoscopic AI was chosen due to its minimally invasive approach and its ability to deposit sperm near the oviducts, thereby improving the likelihood of fertilization.

## Materials and methods

### Animals

A total of five male and six female jaguars from three institutions in Brazil – Associação Mata Ciliar (Jundiaí, São Paulo; *n* = 3 males and 5 females), Parque Bosque dos Jequitibás (Campinas, São Paulo; *n* = 1 male and 1 female), and Zoo Bosque de Pedreira (São Paulo, São Paulo; *n* = 1 male) – participated in this study. Males were housed individually, and females were housed alone or in female–female pairs. All females were housed in proximity to males. Across the three-year study period, male ages ranged from 1.9 to 11.9 years and female ages from 1.8 to 6.1 years. Experiment 1 was conducted between June and October 2016, and the mean female age at the end of sample collection was 3.2 years. In experiment 2, AIs were conducted in April and October 2017. The mean male and female ages at the time of the AIs were 7.3 and 3.9 years, respectively. In experiment 3, AIs were performed in November 2018, and the mean male and female ages at AI were 4.1 and 4.9 years, respectively. For comparison, fecal hormone profiles were also evaluated from two females that conceived naturally and gave birth in April 2017; the mean age at breeding was 2.7 and 5.3 years for males and females, respectively. A detailed summary of individual animals, ages, institutional origin, and participation across experiments is provided in Table 1.

**Table 1:** Ages and locations of study animals. Ages correspond to the end of sample collection (experiment 1), first AI (experiment 2; second AI conducted 6 months later), AI (experiment 3), and breeding (pregnancy profile). The dashes indicate that the animal did not participate in that experiment.

Animal	Institution	Age at experiment (years)			
		Experiment 1	Experiment 2	Experiment 3	Pregnancy profile
Female					
1	AMC	2.9	3.4	5.0	—
2	AMC	3.3	3.8	5.4	—
3	PBdJ	3.5	4.0	5.6	—
4	AMC	2.9	3.4	5.0	—
5	AMC	—	—	3.7	1.8
6	AMC	—	—	—	6.1
Male					
1	AMC	—	2.1	—	1.9*
2	ZBdP	—	11.7	—	—
3	PBdJ	—	11.9	—	—
4	AMC	—	—	5.4	3.5^†^
5	AMC	—	—	2.2	—

* Paired with female 6.

^†^ Paired with female 5.

AMC, Associação Mata Ciliar; PBdJ, Parque Bosque dos Jequitibás; and ZBdP, Zoo Bosque de Pedreira.

All scientific research conducted in Brazil with jaguars was authorized by the Brazil Ministry of Science, Technology, and Innovation, through the National Council for Scientific and Technological Development (MCTI-CNPq certificate number 01300.001870/2015-79), and the Brazil Ministry of the Environment, through the Chico Mendes Institute for Biodiversity Conservation’s Biodiversity Authorization and Information System (SISBIO permit #48853-1). The research was conducted between 2016 and 2018, prior to the implementation of Resolução Normativa no. 51 (May 19, 2021), which introduced the requirement for CEUA approval for foreign scientist-affiliated research carried out in Brazil. Animal use was also approved by and performed in accordance with the policies of the Institutional Animal Care and Use Committee at the Cincinnati Zoo & Botanical Garden.

### Exogenous hormone administration

The synthetic progestin altrenogest oral suspension (Regu-Mate®; 2.2 mg/mL, Intervet Inc., USA) was measured with a syringe and mixed into the cats’ daily food. In experiment 1, four females were randomly assigned to either the low (0.044 mg/kg; *n* = 2) or high (0.088 mg/kg; *n* = 2) treatment group and fed altrenogest daily for 45 days (Table 2, protocol 1).

**Table 2:** Exogenous hormone treatment protocols.

	Protocol 1	Protocol 2	Protocol 3
Altrenogest (mg/kg)	0.044*, 0.088^†^	0.088*, 0.176^†^	0.176
eCG (IU)	—	600	600
pLH (IU)	—	5,000	10,000
Altrenogest treatment (days)	45	45–48	40–44
Altrenogest–eCG interval (days)	—	5	7
eCG–pLH interval (hours)	—	82	90–92
pLH–AI interval (hours)	—	43–48	40–44

* Low dose.

^†^ High dose.

Experiment 2 was a balanced crossover design. Four females were randomly assigned to either low (0.088 mg/kg) or high (0.176 mg/kg) altrenogest treatments, and six months later, each female received the other dose. Females were fed altrenogest daily for 45–48 days and, following a 5-day withdrawal period, treated with exogenous gonadotropins. Lyophilized eCG (ProSpec-Tany TechnoGene Ltd, USA) and pLH (Sioux Biochemical, USA) were reconstituted in sterile water. Individual doses of eCG and pLH were aspirated into 1 mL syringes and kept frozen at −20°C until use (within 2 weeks of freezing). All females received an initial injection of eCG (600 IU), followed by an injection of pLH (5,000 IU) 82 h later. Gonadotropins were administered intramuscularly (IM) via a dart gun. At 43–48 h post-pLH, females were evaluated laparoscopically and assessed for ovulatory response (Table 2, protocol 2).

Females in experiment 3 (*n* = 5) all received the high dose (0.176 mg/kg) of altrenogest. Females were fed altrenogest daily for 40–44 days and, following a 7-day withdrawal period, treated with 600 IU eCG followed by 10,000 IU pLH 90–92 h later. Gonadotropins were administered intramuscularly using a hand-held syringe, with jaguars briefly restrained in a squeeze cage following routine husbandry training to allow voluntary injections. Ovarian response was assessed laparoscopically 40–44 h post-pLH (Table 2, protocol 3).

### Fecal hormone metabolite immunoassays

#### Sample collection, storage, and transport

In experiment 1, fecal samples were collected serially from each female for 3 weeks prior to starting altrenogest treatment, through the duration of treatment, and for 54 days after cessation of treatment. In experiments 2 and 3, samples were collected serially for approximately 30 days prior to altrenogest administration and 110 days following AI. Samples were sealed in plastic bags, labeled with the animal’s name and date collected, and stored at −20°C until lyophilization. After drying, the samples were stored at room temperature and transported to the Cincinnati Zoo and Botanical Garden’s Center for Conservation and Research of Endangered Wildlife (CREW) for endocrine analysis. Sample transport was conducted in 2019 with authorization from the Brazilian Ministry of the Environment and the Genetic Heritage Management Council (CGen; official designation at the time of export, now administered through the National Genetic Heritage and Associated Traditional Knowledge Management System (SisGen)), under Registration No. R09DB04.

Serial sample collections were targeted at three nonconsecutive days per week. However, due to gaps in sample collection frequency, fecal hormone metabolite profiles were only generated for four of eight treatment replicates in protocol 2 and three of five females in protocol 3; all four profiles were generated for protocol 1. In addition, serial fecal samples were collected for comparative analyses from two females during pregnancies that resulted from natural breeding.

#### Sample extraction

Samples were extracted using a previously described method (Herrick *et al.* 2010). Briefly, lyophilized fecal samples were pulverized into a fine powder, and 250 ± 5 mg of each sample were extracted with 2.5 mL of 90% ethanol (1:10 w:v). When 250 mg of fecal powder were not available, the maximum obtainable amount over 50 mg was used, and ethanol was added proportionally. Samples with less than 50 mg of fecal powder were excluded from analysis. The mixture was incubated on a mechanical rocker for at least 18 h. After extraction, samples were centrifuged for 15 min at 1,700 *g*. The supernatants were transferred to 2.0 mL cryovials and stored at −20°C until analysis.

#### Progesterone metabolites assay

Fecal progesterone steroid metabolites (P4) were analyzed with competitive enzyme immunoassay (EIA) using a commercially available progesterone mini-kit (ISWE003, Arbor Assays, USA) following the manufacturer’s instructions. The kit employs a progesterone-specific antibody (CL425) previously validated for use with jaguar fecal samples (Graham *et al.* 2001, Barnes *et al.* 2016). Briefly, sample extracts were diluted in 1x assay buffer (Arbor Assays X065), and 50 μL of samples, standards, and controls were added in duplicate to a 96-well plate pre-coated with goat anti-mouse IgG antibody (Arbor Assays A008). Subsequently, 25 μL of horseradish peroxidase (HRP) conjugate and 25 μL of antibody were added to each well. Each plate included blank wells (no antibody), high control wells (2 pg/μL), and low control wells (0.1 pg/μL) to monitor inter- and intra-assay variability.

Plates were incubated at room temperature on a shaker for 2 h, washed three times with 300 μL of wash buffer (Arbor Assays X007), and developed using 3,3′,5,5′-tetramethylbenzidine (TMB) substrate (Moss). The reaction was stopped after approximately 30 min with 1N hydrochloric acid (HCl), and optical density was measured at 450 nm using a microplate reader (VersaMax Absorbance Microplate Reader, Molecular Devices, USA). Results were recorded using SoftMax Pro 7.0 software.

#### Estradiol metabolites assay

Fecal estradiol steroid metabolites (E2) were analyzed using EIA with a 17β-estradiol-specific antibody (R0008) and HRP-conjugated estradiol, both obtained from Dr C. Munro (Clinical Endocrinology Lab, University of California, Davis, USA), which were previously validated for use with jaguar fecal extracts (Barnes *et al.* 2016). The antibody and HRP conjugate were diluted 1:300,000 in assay buffer (Arbor Assays X065). Sample extracts were diluted in 1× assay buffer, and 50 μL of samples, standards, and controls were added in duplicate to a 96-well plate pre-coated with goat anti-rabbit IgG antibody (Arbor Assays A009). The plate was incubated at room temperature for 1 h, washed three times with 300 μL of wash buffer (Arbor Assays X007), and developed using TMB substrate (Moss). The reaction was stopped after approximately 20 min using 1N HCL. Optical density was measured at 450 nm using a microplate reader, and the results were recorded with SoftMax Pro 7.0 software.

### Semen collection and processing

Semen collections were performed within 2–5 h of AI. Adult male jaguars (*n* = 5) were anesthetized with a combination of ketamine (mean dose, 8.5 mg/kg) and detomidine (mean dose: 50 μg/kg). At 20–30 min post-injection, a urinary catheter was inserted 20 cm into the urethra to recover semen (Zambelli *et al.* 2008, de Araujo *et al.* 2018). Immediately following catheterization, males were subjected to 1–3 series of electroejaculation (EEJ) using a standard technique (Howard 1986) with slight modifications. Aliquots of recovered samples were used to assess sperm motility, rate of forward progression, morphology, and acrosome integrity (Vansandt *et al.* 2021). Semen was then diluted 1:1 to 1:10 with feline optimized culture medium (FOCM) (Herrick *et al.* 2007). Samples containing motile sperm were pooled, and sperm concentration was determined using a hemocytometer. Sperm samples were maintained in the dark at room temperature until the AI procedure. Immediately before the AI, the diluted semen was centrifuged for 8 min at 600 *g* and the resulting sperm pellet was suspended in FOCM to a total volume of 20 μL (75–220 × 10^6^ motile sperm/mL) for oviductal AI or 60 μL (50–210 × 10^6^ motile sperm/mL) for uterine AI.

### Laparoscopic AI procedure

Female jaguars were anesthetized with an IM combination of ketamine (mean dose: 8.2 mg/kg) and detomidine (mean dose: 80 μg/kg) and maintained on 0.5–2% isoflurane delivered via an endotracheal tube. Laparoscopy was performed as previously described (Conforti *et al.* 2013), with minor modifications. The female was positioned in dorsal recumbency, and a surgical scrub of the abdominal region was performed. After the jaguar was placed in the Trendelenburg position, a 10 mm laparoscope (Olympus Corporation, Japan) attached by a fiber optic cable to an Olympus light source was introduced through the midline abdominal wall (approximately halfway between the xiphoid and umbilicus) to allow visualization of the abdominal cavity. Before AI, the reproductive tract was evaluated for any potential pathology or abnormalities. Ovarian follicles (≥2 mm diameter) and corpora hemorrhagica (CH) were counted and measured, and the uterine horns were assessed for diameter, tone, and the presence/absence of segmentation.

For laparoscopic oviductal artificial insemination (LOAI), an accessory 5 mm trocar-cannula was introduced through the abdominal wall (approximately 1 cm right lateral of the umbilicus) and custom-made grasping forceps (MDS Incorporated, USA) were inserted to secure the fimbriae at the craniomedial edge of the ovarian bursa. The bursa was extended and everted to allow visualization of the abdominal oviductal ostium. A 16-gauge 1¼-inch intravenous catheter was inserted percutaneously caudolateral to the left ovary. A blunt 20-gauge 4-inch needle (Hamilton Company, USA) was pre-loaded with 10 μL of concentrated sperm at the distal end. The needle was attached to a 1 mL syringe and inserted through the catheter into the abdominal ostium. Sperm was deposited into the oviductal lumen with slight air pressure from the syringe. The procedure was repeated on the other oviduct.

If the oviduct could not be cannulated, a laparoscopic uterine AI (LUAI) was performed as previously described (Howard *et al.* 1992). Atraumatic grasping forceps (Richard Wolf Medical Instruments Corporation, USA) were inserted into the accessory cannula and used to secure the uterine horn against the ventral abdominal wall. A 16-gauge, 2-inch intravenous catheter was inserted through the abdominal wall into the uterine lumen near the ovarian end of the uterine horn. A pre-loaded blunt 20-gauge 4-inch needle attached to a 1 mL syringe was passed through the catheter into the uterine lumen, and slight air pressure from the syringe was used to deposit sperm into the lumen.

### Statistical analysis

Hormone baseline values were calculated for each female during each experimental phase or pregnancy using the R statistical package hormLong (version 1.0, Fanson & Fanson (2015)). Baseline values were determined for each individual through an iterative process, excluding all points greater than the mean plus 1.5 standard deviations (SDs) for E2 and 2.0 SDs for P4 (Graham *et al.* 2000, Pelican *et al.* 2005, Herrick *et al.* 2010). An estrus phase was defined as two or more consecutive samples with values greater than the E2 baseline plus 1.5 SDs. Similarly, a luteal phase was defined as six or more consecutive samples exceeding the P4 baseline mean plus 2 SDs.

All remaining analyses were conducted within the MIXED procedure using SAS® Studio software, Release: 3.81, Enterprise Edition (SAS Institute Inc., USA). For experiment 1 (control vs treated, low dose vs high dose altrenogest), E2 concentrations were compared using a generalized linear mixed model, with treatment group, period, and the treatment group × period interaction term as fixed effects and individual as a random factor. Follicular diameter, the total number of follicles + CH, ovulation rate (percent of follicles that ovulated), and the total number of ovulations were compared using a generalized linear mixed model, with treatment as a fixed effect and individual as a random factor. For follicular diameter, due to a significant lack of homogeneity of variances between individuals, independent variances were calculated and used for each animal. Similarly, due to a significant lack of homogeneity of variances between treatments for ovulation rate and the total number of ovulations, independent variances were calculated (by treatment group) and used. Corpus hemorrhagicum diameter was compared using a post hoc contrast due to the incomplete factorial structure of the generalized linear model (since not all females ovulated with each treatment), with treatment and individual as fixed factors.

When comparing outcomes from using protocol 2 vs protocol 3, the total number of follicles and CH, ovulation rate, and the total number of ovulations were compared using a generalized linear mixed model, with protocol as a fixed effect and individual as a random factor. Due to a significant lack of homogeneity of variances between protocols for all three outcomes measured, independent variances were calculated (by protocol) and used.

## Results

### Effect of oral progestin dosage on ovarian cyclicity

**Figure 1:**
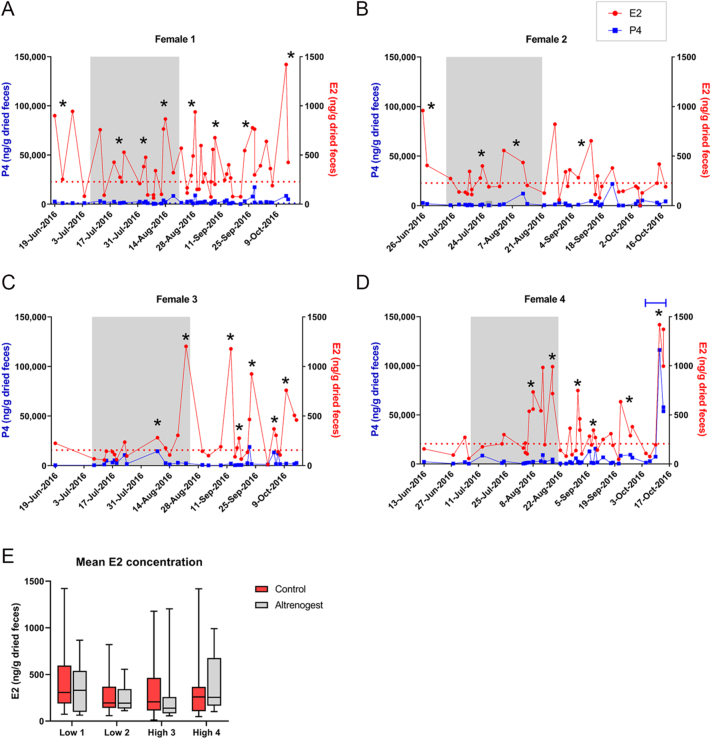
Fecal hormone metabolite profiles before, during, and after altrenogest administration with Protocol 1. Profiles include (A and B) low (0.044 mg/kg) and (C and D) high (0.088 mg/kg) treatment groups. Black asterisk indicates an estrus phase; solid blue line indicates luteal phase. Red dotted line represents E2 peak cut-off value (baseline mean + 1.5 SD) and area within gray bar indicates altrenogest treatment period. (E) Box and whisker plot of mean E2 concentration during altrenogest treatment as compared to the non-treated period (Control). Treatment group and female number are indicated on X-axis.

In experiment 1, adult female jaguars were fed altrenogest at two doses (low: 0.044 mg/kg, *n* = 2; high: 0.088 mg/kg, *n* = 2) daily for 45 days (Table 2, protocol 1). Longitudinal fecal hormone analyses revealed that neither dose was sufficient to suppress ovarian activity. In the low-dose treatment group, females 1 and 2 exhibited three and two estrus phases, respectively, during the treatment period (Fig. 1A and B). In the high dose group, both females displayed two estrus phases (Fig. 1C and D). There was no difference in mean E2 concentration during altrenogest treatment as compared to the non-treated portion of the observation period (Fig. 1E, *P* = 0.6405) and no significant differences between treatment groups (*P* = 0.9685). No spontaneous ovulations were observed during altrenogest treatment, but a female in the high-dose treatment group exhibited one spontaneous ovulation (defined as a luteal phase that occurs without natural mating or exogenous gonadotropin treatment preceding it) at the end of the observation period (Fig. 1D, blue bar).

### Impact of exogenous hormone treatments on ovarian follicular development

In experiment 2, four females were randomly assigned to either low or high altrenogest treatments, and six months later, each female received the other dose. Based on the results of experiment 1, the altrenogest dose was doubled for each treatment group in protocol 2 (Table 2). Reproductive tracts were assessed at the time of laparoscopic AI. Uterine horn diameters ranged from 12 to 18 mm (mean, 15.7 ± 0.8). One female had slight uterine tone (female 1, low treatment); the remaining three females had moderate tone. No uterine horn segmentation was noted for any female.

**Figure 2:**
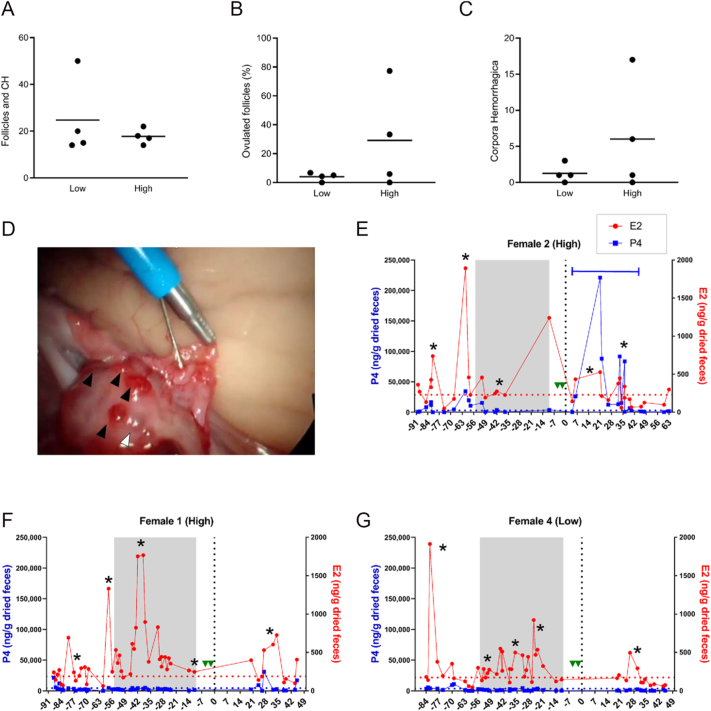
Effects of Protocol 2 on ovarian response and hormone profiles. Scatter plots comparing (A) total number of mature follicles and corpora hemorrhagica, (B) percent of ovulated mature follicles, and (C) number of corpora hemorrhagica between low and high treatment groups in Protocol 2, as determined by laparoscopic examination of ovaries at the time of artificial insemination. Each dot represents an individual animal, with horizontal bars indicating group means. (D) Laparoscopic image of cannulation of the right oviduct in Female 2 (high treatment). Black arrowheads indicate fresh corpora hemorrhagica and white arrowhead indicates a mature follicle. Fecal hormone metabolite profiles for Females (E) 2 (high treatment with multiple ovulations), (F) 1 (high treatment with no ovulations), and (G) 4 (low treatment with one ovulation). Black asterisk indicates estrus phase; solid blue line indicates luteal phase. Red dotted line represents E2 peak cut-off value (baseline mean + 1.5 SD), blue dotted line represents P4 peak cut-off value (baseline mean + 2 SD), and vertical black dotted line indicates time of laparoscopic AI. Green triangles indicate eCG and pLH treatments, and area within gray bar indicates altrenogest treatment period.

All females developed multiple mature follicles in response to protocol 2 and most females (3 of 4 in each treatment group) had at least one ovulation (Table 3, CH). Follicle diameter ranged between 2 and 6 mm (mean, 3.5 ± 0.2) in the low-dose group and between 4 and 10 mm (mean, 5.6 ± 0.2) in the high-dose group and did not differ between treatments (*P* = 0.1970). Corpus hemorrhagicum diameter ranged between 4 and 6 mm (mean, 5.2 ± 0.5) in the low-dose group and between 2 and 10 mm (mean, 5.3 ± 0.2) in the high-dose group and also did not differ between treatments (*P* = 0.7139). There was no difference between low-dose and high-dose treatment groups for the combined number of mature follicles and CH (Fig. 2A, *P* = 0.4632), percent of follicles that ovulated (Fig. 2B, *P* = 0.25), or the number of CH observed (Fig. 2C, *P* = 0.7139). In addition, there was a high level of individual variation in the rate of ovulation (i.e., the number of CH/the total number of mature follicles plus CH) for both treatment groups (Table 3).

**Table 3:** Protocol 2 laparoscopic AI results.

Female	Tx	Follicles	CH	Ovulation rate (%)	LP (days)	Sperm sample*	Left side		Right side	
							AI technique	Motile sperm (× 10^6^)	AI technique	Motile sperm (× 10^6^)
1	L	11	3	21.4	S	A	O	2.5	O	2.5
2	L	19	1	5.0	34	B	O	2.5	O	2.5
3	L	50	0	0.0	S	C	O	4.2	U	12.6
4	L	14	1	6.7	0	D	O	1.5	U	3
1	H	14	0	0.0	0	D	U	4	U	4
2	H	5	17	77.3	S	E	O	3.4	O	3.4
3	H	12	6	33.3	S	F	U	10	O	2.5
4	H	16	1	5.9	0	A	O	2.5	O	2.5

* See Table 4.

Tx, altrenogest treatment group; L, low altrenogest dose; H, high altrenogest dose; CH, Corpora hemorrhagica; LP, length of luteal phase following hormone treatment protocol, as determined by 6+ consecutive samples with P4 above baseline plus 2 SDs; S, not enough fecal samples to analyze LP; O, oviductal AI; U, uterine AI.

For females with adequate number of fecal samples to perform hormone metabolite analysis (*n* = 4), progestin profiles generally conferred with visual ovarian assessments. A sustained rise in P4 was observed in female 2 (high treatment), which had 17 ovulations at the time of AI (Table 3, Fig. 2D and E), whereas no rise in P4 was present in female 1 (high treatment), which had 0 ovulations (Table 3, Fig. 2F). However, female 4, which had a single ovulation at each AI, did not demonstrate a concurrent rise in P4 following either low or high treatment (Table 3, Fig. 2G).

For the AIs, ovulating females were inseminated in each oviduct with fresh semen (1.5–4.2 × 10^6^ motile sperm/oviduct) from resident jaguar males collected on the morning of the respective procedure (Tables 3 and 4). If the oviduct could not be cannulated (*n* = 5 oviducts total, involving 4 females), fresh semen was deposited into the ipsilateral uterine horn (3–12.6 × 10^6^ motile sperm/horn). No births resulted from the AIs conducted using protocol 2.

**Table 4:** Semen quality characteristics of male jaguars used for protocol 2 AIs.

Sperm sample*	Male	Date of collection	Total sperm (× 10^6^)	T mot (%)	RFP (0–5)	Acrosome (% intact)	Morphology (% normal)	Major abnormality (% of sperm)
A	1	October 24, 2017	177.8	80	3.0	97	86	Abnormal midpiece (3%)
B	2	October 23, 2017	159.6	70	3.0	83	52	Bent tail (22%)
C	3	April 09, 2017	77.0	70	3.5	90	26	Bent midpiece with droplet (29%)
D	1	April 08, 2017	60.9	80	3.0	92	75	Bent tail (7%)
E	2	April 06, 2017	24.1	80	3.5	88	42	Proximal droplet (17%)
F	3	October 27, 2017	390.3	80	3.5	95	55	Bent midpiece with droplet (14%)

* See Table 3.

T mot, total sperm motility; RFP, rate of forward progression.

### Refinement of hormonal treatment to enhance ovulation and artificial insemination success

In experiment 3, five females were assigned to hormone treatment protocol 3 (Table 2). Three adjustments were made compared to protocol 2: i) the progestin withdrawal period was increased from 5 to 7 days, ii) the eCG–pLH interval was increased from 82 h to 90–92 h, and iii) the pLH dose was increased from 5,000 to 10,000 IU. Reproductive tracts were assessed at the time of laparoscopic AI. Uterine horn diameters ranged from 14 to 16 mm (mean: 14.8 ± 0.5). Female 1 had slight uterine horn tone and no uterine horn segmentation; the remaining four females had moderate tone and slight segmentation.

**Figure 3:**
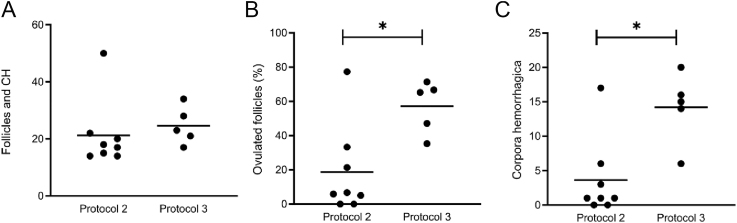
Comparison of ovarian responses between Protocols 2 and 3. Scatter plots comparing (A) total number of mature follicles and corpora hemorrhagica, (B) percent of ovulated mature follicles, and (C) number of corpora hemorrhagica, as determined by laparoscopic examination of ovaries at the time of artificial insemination. Each dot represents an individual animal, with horizontal bars indicating group means. *P ≤ 0.05.

All females developed multiple mature follicles and CH in response to protocol 3 (Table 5). Follicle diameter ranged between 2 and 6 mm (mean, 4.5 ± 0.2). Corpus hemorrhagicum diameter ranged between 4 and 6 mm (mean: 4.6 ± 0.1). The combined number of mature follicles and CH did not differ from protocol 2 (Fig. 3A, *P* = 0.3732). However, the percentage of mature follicles that ovulated was significantly higher with protocol 3 (57.1 ± 6.8%) compared to protocol 2 (17.0 ± 9.9%, Fig. 3B, *P* = 0.0084). In addition, the total number of ovulations was significantly greater in protocol 3 (14.2 ± 2.3) than in protocol 2 (3.6 ± 2.0, Fig. 3C, *P* = 0.0107).

**Table 5:** Protocol 3 laparoscopic AI results.

Female	Follicles	CH	Ovulation rate (%)	LP (days)	Sperm sample*	Left side		Right side	
						AI technique	Motile sperm (× 10^6^)	AI technique	Motile sperm (× 10^6^)
1	8	20	71.4	47	G	U	5	O	2.5
2^†^	7	14	66.7	112	H	O	2.5	U	7.5
3	18	16	47.1	S	I	O	4.4	O	4.4
4	11	6	35.3	26	G	O	2.5	O	2.5
5	8	15	65.2	S	G	O	2.5	O	2.5

* See Table 6.

^†^ Female that gave birth.

CH, Corpora hemorrhagica; LP, length of luteal phase following hormone treatment protocol, as determined by 6+ consecutive samples with P4 above baseline plus 2 SDs; S, not enough fecal samples to analyze LP; O, oviductal AI; and U, uterine AI.

For the AIs, ovulating females were inseminated in each oviduct with fresh semen (2.5–4.4 × 10^6^ motile sperm/oviduct) from resident jaguar males collected on the morning of the respective procedure (Tables 5 and 6). If the oviduct could not be cannulated (*n* = 2 oviducts total, involving 2 females), fresh semen was deposited into the ipsilateral uterine horn (5–7.5 × 10^6^ motile sperm/horn).

**Table 6:** Semen quality characteristics of male jaguars used for protocol 3 AIs.

Sperm sample*	Male	Date of collection	Total sperm (× 10^6^)	T mot (%)	RFP (0–5)	Acrosome (% intact)	Morphology (% normal)	Major abnormality (% of sperm)
G	4	November 06, 2018	97.8	80	4.0	95	67	Proximal droplet (14%)
H	5	November 05, 2018	49.4	80	4.0	98	63	Proximal droplet (15%)
I	5	November 09, 2018	47.1	70	3.5	93	71	Bent tail (17%)

* See Table 5.

T mot, total sperm motility; RFP, rate of forward progression.

**Figure 4:**
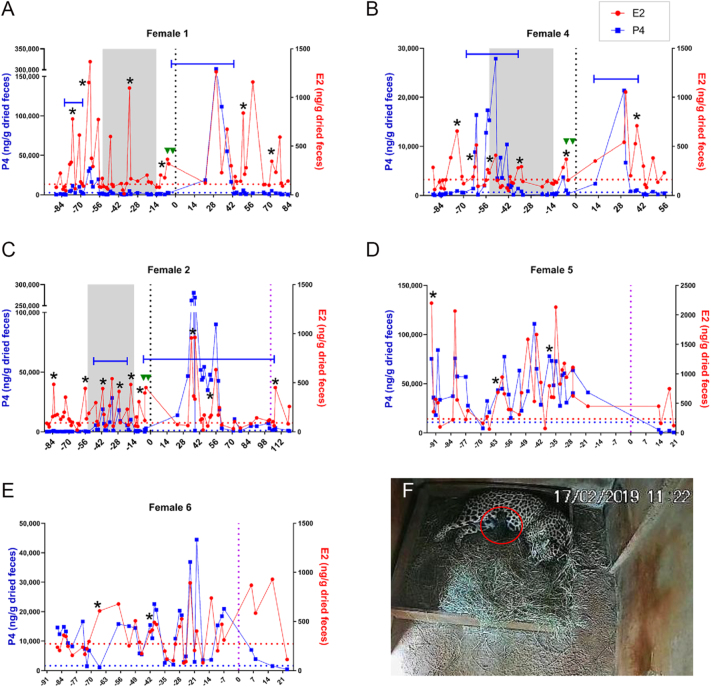
Effects of Protocol 3 on fecal hormone profiles and pregnancy outcomes. Fecal hormone metabolite profiles for Females (A) 1, (B) 4, and (C) 2, that all responded to Protocol 3 with multiple fresh ovulations at the time of AI and a sustained rise in P4 following the procedure. Fecal hormone metabolite profiles for two additional females (D and E) during pregnancies that resulted from natural breeding for comparison. Black asterisk indicates an estrus phase; solid blue line indicates a luteal phase. Red dotted line represents E2 peak cut-off value (baseline mean + 1.5 SD), blue dotted line represents P4 peak cut-off value (baseline mean + 2 SD), and vertical black dotted line indicates time of laparoscopic AI. Green triangles indicate eCG and pLH treatments, and area within gray bar indicates altrenogest treatment period. Vertical purple dotted line in (C–E) indicates day of birth. (F) Remote monitoring image of Female 2 and her cub (circled in red) nursing, one day after birth.

Fecal hormone metabolite analysis was performed for females with adequate sample collection frequency (*n* = 3). All three females showed a sustained rise in P4 following observation of multiple CH at the time of AI (Table 5, Fig. 4A, B, C). Two representative profiles from female jaguars that gave birth following natural matings are included for comparison (Fig. 4D and E). Female 2 gave birth to a single live cub 103 days post-AI (Fig. 4F). The cub appeared vigorous and showed active nursing behavior under remote camera monitoring for two days post-birth, but disappeared on the third day, and presumably was consumed by the dam. No other births resulted from protocol 3 AIs.

## Discussion

The first successful AI in the domestic cat was reported in 1970 (Sojka *et al.* 1970). In the 55 years that have ensued, successful AIs have been reported in 14 wild felid species (reviewed by Swanson (2023)). In the six cat species comprising the genus *Panthera*, full-term offspring have been produced by AI in the lion (*P. leo*) (Callealta *et al.* 2019), tiger (*P. tigris*) (Donoghue *et al.* 1993, Chagas e Silva *et al.* 2000, Lambo *et al.* 2014), leopard (*P. pardus*) (Dresser *et al.* 1982, Lueders *et al.* 2015), and snow leopard (*P. uncia*) (Roth *et al.* 1997). However, this is the first report of a successful pregnancy and birth resulting from AI in the jaguar, the final member of the pantherine lineage to achieve that scientific landmark.

Before considering the broader implications of this work, it is important to acknowledge the limitations of the study. First, as with many studies involving wildlife, access to a large number of reproductively suitable jaguars is limited due to ethical, logistical, and regulatory constraints. As a result, sample sizes were small, and most females were enrolled in multiple phases of the study to maximize data collection. Four of the five females (females 1–4) participated in all three experiments, enabling within-animal comparisons across protocol refinements, while one female (female 5) took part only in experiment 3. Although this repeated-measures approach allowed meaningful, stepwise improvement of the protocols, caution should be applied when extrapolating the findings to the species as a whole, as individual variation may influence responsiveness. Second, fecal hormone metabolite monitoring yielded useful information, but incomplete sampling limited the resolution and number of endocrine profiles. Future studies incorporating more frequent sampling would help strengthen endocrine assessments.

One of the major challenges of AI in any felid species is ensuring that the procedure is performed during the appropriate stage of the female’s estrous cycle (reviewed by Thongphakdee *et al.* (2020)). Historically, felid ovulation synchronization protocols have utilized a combination of eCG and human chorionic gonadotropin (hCG) to induce follicular maturation and ovulation, respectively (reviewed by Pelican *et al.* (2006)). However, both are large glycoproteins with extended circulatory persistence in felids (4–5 days in the domestic cat), which can induce anti-gonadotropin antibody formation and cause the female to become refractory to future treatments (Swanson *et al.* 1995, 1996*a*, 1997). Because hCG also has folliculogenic activity, it promotes ancillary follicles and secondary corpora lutea (Swanson *et al.* 1996*b*), which can disrupt the post-ovulatory endocrine environment and have a negative impact on embryo survival following AI or embryo transfer (ET) (Graham *et al.* 2000). Alternatively, pLH has a very short half-life and only remains in circulation for hours following injection (Ascoli *et al.* 1975). It has proven to be highly effective for use in ovarian synchronization protocols with eCG to induce ovulation and produce high pregnancy percentages in ET and AI procedures in the domestic cat (Magarey *et al.* 2005, Conforti *et al.* 2013). A similar eCG/pLH ovulation induction protocol has subsequently produced AI pregnancies in the sand cat (*Felis margarita*), leopard cat (*Prionailurus bengalensis*), Pallas’ cat (*Otocolobus manul*), fishing cat (*Prionailurus viverrinus*), ocelot (*Leopardus pardalis*), clouded leopard (*Neofelis nebulosa*), and tiger (Swanson 2012, 2016, 2017*a*, 2023, Lambo *et al.* 2014, Tipkantha *et al.* 2017, Azumano *et al.* 2022).

It is important to note that gonadotropin doses for domestic cats cannot simply be extrapolated to wild felids based on body weight. Instead, species-specific variations in ovarian sensitivity to hormonal treatment must be carefully considered. For instance, members of the *Leopardus* lineage, including the ocelot and southern tigrina (*Leopardus guttulus*), are relatively insensitive to gonadotropins and require higher doses compared to species such as the cheetah (*Acinonyx jubatus*) or clouded leopard, which exhibit greater sensitivity despite their larger body size (Swanson & Brown 2004, Pelican *et al.* 2006).

Efforts to apply ART in jaguars have explored several gonadotropin-based ovarian stimulation strategies, but with limited reproductive success. Two studies evaluated jaguar ovarian responses to eCG (600–800 IU) with or without hCG (300–500 IU) for oocyte recovery (Barnes *et al.* 2016, Jorge-Neto *et al.* 2023). Ovulation response varied by protocol, timing of assessment, inclusion of hCG, and/or exposure to a male (proximity or same-pen housing), as determined via fecal progestin profile (Barnes *et al.* 2016) or laparoscopic ovarian evaluation (Jorge-Neto *et al.* 2023). Both studies reported recovery of morphologically high-quality oocytes. However, *in vitro* fertilization (IVF) was not attempted, and thus, oocyte developmental competence remains unknown. Morato *et al.* (2002) tested a multiple-dose porcine follicle stimulating hormone regimen followed by a single pLH treatment in three jaguars, resulting in six cleaved embryos (2–4 cell stage) from 26 oocytes fertilized by IVF. Embryo transfers were not performed, so subsequent developmental potential could not be assessed. The only reported attempt at laparoscopic AI in jaguars used an eCG/hCG stimulation protocol in three females: one showed no follicular response, one had an ovarian tumor identified at the time of insemination, and one ovulated and was inseminated but did not conceive (Jimenez *et al.* 1999). To the authors’ knowledge, there are no previous reports of successful ovulation induction in the jaguar utilizing eCG and pLH (reviewed by de Deco-Souza *et al.* (2024)).

Another unique aspect of felid reproductive physiology is that many cat species, including the jaguar, were classically categorized as induced ovulators with the act of copulation serving as the canonical stimulus (Wildt *et al.* 1979, Brown 2011). It should therefore be easy to manipulate a jaguar’s estrous cycle because, in the absence of mating, the female would ostensibly lack corpora lutea and not require luteal control protocols. However, fecal progestin metabolite analysis by Barnes *et al.* (2016) found that three jaguar females housed in proximity to, but not in direct contact with males, spontaneously ovulated, as evidenced by a sustained elevation in fecal progestin metabolites without induction by copulation. In contrast, none of the four control females housed without male proximity exhibited spontaneous ovulation. Similarly, Jorge-Neto *et al.* (2020) observed a comparable pattern in jaguars stimulated with eCG and evaluated 3–5 days later via laparoscopy: two of the four females with visual, olfactory, and/or auditory contact with males ovulated, whereas both females housed in complete isolation from males did not. These findings suggest that sensory contact with a male either is necessary or greatly enhances the likelihood of spontaneous ovulation during natural and induced estrus periods. Consistent with this conclusion, results from our study also documented spontaneous ovulation in three female jaguars exposed to visual, olfactory, and auditory exposure from a male without any direct physical contact: female 1 (Fig. 4A), female 2 (Fig. 4C), and female 4 (Figs 1D and 4B).

The jaguar’s moderate tendency for spontaneous ovulation poses a challenge to developing successful ovulation induction protocols because endogenous progesterone can blunt the ovarian responsiveness to exogenous gonadotropin treatment and impede fertilization (Pelican *et al.* 2006). Even in the absence of progesterone, endogenous gonadotropins can disrupt the ovary’s ability to respond effectively to hormone treatments. Research across multiple mammalian species has demonstrated that a quiescent ovary responds more uniformly and predictably to exogenous gonadotropin stimulation (Foxcroft 1999, Ron-El *et al.* 2000). In this state, the ovary contains only primordial follicles and follicles in a gonadotropin-independent continuous growth phase, resulting in a population of early antral follicles that are highly receptive to stimulation (McGee & Hsueh 2000). By hormonally stimulating an inactive ovary, these early antral follicles are synchronously recruited for maturation and ovulation (Pelican *et al.* 2006).

To achieve ovarian quiescence prior to gonadotropin treatment, we evaluated the oral progestin altrenogest, which has been successfully used to downregulate ovarian activity in domestic cats (Stewart *et al.* 2010), cheetahs (Crosier *et al.* 2017), and tigrinas (Micheletti *et al.* 2015). In the previous domestic cat study, three altrenogest doses were tested, with the intermediate dose (0.088 mg/kg) proving most effective. This dose maintained normal baseline estrogen and progesterone levels while facilitating a more consistent return to follicular activity versus the low-dose (0.044 mg/kg) or high-dose (0.352 mg/kg) treatment. In protocol 1 (Table 2), altrenogest treatment was administered for 45 days, which corresponds to the approximate mean length of a non-pregnant luteal phase in the jaguar (Barnes *et al.* 2016, Gonzalez *et al.* 2017). The treatment interval was designed to support the natural regression of any existing corpora lutea and prevent the occurrence of new spontaneous ovulations. We chose to investigate the low and intermediate doses of altrenogest, based on findings in other neotropical felids. Our previous studies demonstrated that both the ocelot and the southern tigrina achieve effective ovarian suppression at a dose of 0.044 mg/kg (Lambo *et al.* 2014; unpublished data). In other wild felid studies (Micheletti *et al.* 2015, Crosier *et al.* 2017), higher altrenogest dosages (0.088 mg/kg, cheetah; 0.192 mg/kg, tigrina) were used for short-term (7–14 days) periods of ovarian suppression. In the current study, fecal hormone data revealed that neither the low (0.044 mg/kg) nor high (0.088 mg/kg) dose was sufficient to suppress ovarian cyclicity in the jaguar (Fig. 1A, B, C, D). In addition, mean E2 concentrations showed no difference before and during altrenogest treatment (Fig. 1E).

Based on these findings, we elected to double the altrenogest doses for protocol 2 (Table 2). However, even at the highest dose (0.176 mg/kg), females continued to exhibit follicular activity (Fig. 2E and F). The decision to not further increase the altrenogest dose was based on two lines of reasoning. First, in the domestic cat, ovulation following natural breeding is an all or nothing phenomenon; once the threshold level of LH is reached, all mature follicles ovulate, irrespective of the number of breeding events (Wildt *et al.* 1980). The jaguars in protocol 2 demonstrated a wide range of ovulation rates (0–77.3%), with only three females exceeding 20 percent (Table 3). In addition, one female failed to form a functional corpus luteum with either the low- or high-dose treatment in protocol 2 (based on P4), despite visual confirmation of one fresh ovulation during laparoscopy (Table 3, female 4). While the cause of ovulation/luteinization failure is likely multifactorial, progestin treatment may have contributed via impaired follicular maturation. Second, despite the persistence of follicular activity during altrenogest treatment, no spontaneous ovulations were observed. The domestic cat can demonstrate follicular activity during a luteal phase (Wildt *et al.* 1981), and similarly, our fecal hormone analysis of two jaguars during pregnancy following natural breeding also revealed follicular activity during their luteal phases (Fig. 4D and E). These data suggest that complete ovarian inhibition may not be possible, necessary, or desirable in the jaguar prior to gonadotropin stimulation.

Accordingly, in protocol 3 (Table 2), three adjustments were made to the ovarian synchronization regimen to attempt to optimize ovulatory responses and potential fertility with AI. Although the altrenogest dose was maintained at 0.176 mg/kg, the altrenogest withdrawal period was extended from five to seven days to give follicles additional time to recover from progestin suppression. In addition, the interval between eCG and pLH treatment was lengthened from 82 to 90–92 h, providing maturing follicles more time to gain competency to respond effectively to pLH stimulation. Finally, the pLH dose was increased from 5,000 to 10,000 IU to enhance circulatory persistence and ovulation stimulation over time. Rather than pursuing full ovarian inhibition, protocol 3 sought a level of suppression sufficient to minimize spontaneous ovulation while supporting adequate follicular recovery to preserve ovarian responsiveness to gonadotropins, which may represent a more effective balance for this species.

Collectively, these changes led to an increased ovulatory rate (35.3–71.4%, Table 5), albeit not the 100% rate observed in naturally bred domestic cats (Wildt *et al.* 1980). Notably, all five females treated with this protocol were successfully induced to ovulate, and of the three females that fecal hormone metabolite profiles could be generated, all demonstrated a sustained luteal phase following ovulation induction (Table 5 and Fig. 4A, B, C). Of tantamount importance, one of the five females (female 2) conceived following AI and, following a 103-day gestation period, gave birth to a live cub (Fig. 4C and F). Remote video monitoring showed excellent maternal care and nursing by a vigorous cub on the first day following birth. Unfortunately, the cub disappeared from the maternity den two days after birth, presumably consumed by the mother. While disappointing that the cub did not survive, maternal consumption of offspring is not uncommon among carnivores, first-time mothers, or animals under human care (reviewed by Packer & Pusey (1984)).

Another key challenge with ART in felids has been the large number of sperm required for AI. Empirically, the most successful technique for AI is laparoscopic uterine AI (LUAI), where sperm are surgically deposited into the uterus through small abdominal incisions. Uterine AI often requires large numbers of sperm (>10 million motile), which are not always available from felids (Swanson 2019). While this method has produced offspring in 11 wild cat species, its efficiency remains low, with pregnancy rates typically below 10% with freshly collected sperm (reviewed by Howard & Wildt (2009)). The use of frozen–thawed sperm has resulted in even lower AI success, with only five pregnancies ever produced in wild cats by laparoscopic uterine AI (one leopard cat, one ocelot, and three cheetahs), which precludes its use as a reliable tool for population management.

To improve procedural outcomes, we previously developed a laparoscopic oviductal AI (LOAI) technique, hypothesizing that placing sperm closer to the fertilization site (oviduct vs uterus) could achieve higher pregnancy rates with fewer motile sperm. Results in domestic cats confirmed the superiority of LOAI (56% pregnancy) over LUAI (38%) with low sperm numbers (Conforti *et al.* 2013). Our subsequent studies have demonstrated that LOAI with frozen–thawed domestic cat sperm can result in high pregnancy percentages and normal litter sizes, with fertility equal to that of freshly collected semen (Lambo *et al.* 2012, Swanson *et al.* 2014, 2019). To date, eCG/pLH treatment and LOAI have resulted in multiple term pregnancies in five small cat species (sand cat, leopard cat, Pallas’ cat, fishing cat, and ocelot) and two large cat species (clouded leopard and tiger) (Swanson 2012, 2016, 2017*a*, 2023, Lambo *et al.* 2014, Tipkantha *et al.* 2017, Azumano *et al.* 2022). In the tiger, pregnancy resulted from insemination with only 150,000 motile sperm, highlighting the minimal sperm numbers needed to achieve fertilization.

Performing LOAI in the domestic cat is relatively straightforward, due to the ease of visualizing the abdominal ostium and cannulating the oviduct (Swanson 2019). In contrast, LOAI in jaguars poses significant challenges. Their larger body size complicates transport to the veterinary hospital and makes it more difficult to position them safely in the Trendelenburg position. Furthermore, the higher amount of intra-abdominal fat in jaguars hinders laparoscopic visualization, and anatomical differences (e.g., short oviductal fimbriae and constricted ovarian bursa) further obstruct access to the oviduct for successful cannulation. In experiment 2, cannulation was unsuccessful in five oviducts out of 16 attempts, and only one female (female 2) had both oviducts successfully cannulated during both LOAI procedures (Table 3). Females whose oviducts could not be cannulated on one or both sides likely had a different fertilization potential than those in which LOAI was achieved bilaterally. As a result, fertility outcomes in experiment 2 were influenced not only by the hormonal treatment protocol (low vs high altrenogest) but also by the insemination method used, and this should be considered when interpreting the results.

This distinction is also important when evaluating outcomes in experiment 3, in which cannulation was unsuccessful in one oviduct for two different females (Table 5). Notably, one of these jaguars was female 2, which gave birth to a single cub. Consequently, it remains uncertain whether fertilization occurred from the oviductal or uterine insemination. However, considering the higher LOAI success rates observed in domestic cats, the relatively low number of motile sperm (7.5 × 10^6^) inseminated into this female’s uterine horn, and the greater number of ovulations on the oviductal insemination side (10 CH) compared to the uterine insemination side (4 CH), it is probable that the LOAI procedure was responsible for the successful pregnancy.

## Conclusion

This study represents a critical milestone in jaguar reproductive research, marking the first successful pregnancy achieved through artificial insemination in this species. Jaguars present distinct reproductive physiological challenges, including a moderate tendency for spontaneous ovulation, variability in ovarian sensitivity to exogenous hormone treatment, and anatomical differences that complicate assisted reproductive procedures. These challenges necessitate tailored approaches to hormone treatment protocols and AI techniques, emphasizing the importance of understanding species-specific differences when adapting ART from model species, such as the domestic cat, to wildlife. Through iterative adjustments to hormonal treatments, we developed a protocol that successfully induced ovulation in all treated jaguars, culminating in a live birth following laparoscopic AI. Despite the ultimate loss of the cub, this study demonstrates the feasibility of applying ART to jaguars and underscores the importance of continued refinement of reproductive protocols and novel strategies to overcome species-specific physiological and logistical challenges. The small number of females available for this research limits the ability to broadly generalize reproductive responses across the species; however, the stepwise improvements observed provide a promising foundation for further optimization. Future studies involving additional females across more diverse ages, genetic backgrounds, and institutions will be important to further validate and optimize this approach. With continued refinement, we expect that ART may play a pivotal role in ensuring the long-term sustainability of jaguar populations, both under human care and in the wild.

## Declaration of interest

All authors declare that there is no conflict of interest that could be perceived as prejudicing the impartiality of the research reported.

## Funding

This research was supported by internal institutional funding from the Associação Mata Ciliar and the Cincinnati Zoo and Botanical Garden.

## Author contribution statement

LMV, CAH, PRY, RCRdP, HLB, and WFS conceived and designed the experiments. LMV, CAH, PRY, JdSP, RCRdP, HLB, and WFS conducted data collection. HLB and EMD performed ELISA measurements of fecal hormone metabolites. EMD conducted statistical analyses of fecal hormone data, while LMV completed all other statistical analyses. LMV drafted the original manuscript, which was edited by EMD and WFS. All authors critically reviewed and approved the final manuscript.
